# Current status of transcriptome sequencing technology in ruminants

**DOI:** 10.3389/fvets.2025.1558799

**Published:** 2025-06-26

**Authors:** Cui JiaYu, Song Lili, Wang Dawei, Liu ZhiLin, Zhang Xin, Jia Zelin, Zhang Yuhang, Xiong Huisheng, Wang Xueli

**Affiliations:** ^1^College of Animal Science and Technology, Inner Mongolia Minzu University, Tongliao, China; ^2^Department of Grassland Ecology and Animal Husbandry Veterinary Medicine, Xilingol Vocational College, Xilingol League, China; ^3^Tongliao Animal Disease Prevention and Control Center, Tongliao, China

**Keywords:** RNA seq, ruminant animals, application status, lactation, reproduction, meat quality, disease

## Abstract

In many parts of the world, safe ruminant production underpins food security, while ruminant meat and milk are important agricultural commodities and a major source of protein requirements in the human diet. In order to maintain the sustainability of such agricultural products, animal production should be made more efficient through better management and production techniques. Ruminating animals such as cows and sheep have been used for the synthesis of dairy products, the production of high-quality meat, and the study of reproductive mechanisms. Using transcriptome technology in ruminant ecosystems has sped up the study of animal diversity under various feeding and production conditions. These studies have provided sufficient information to reduce farm pollution and improve farming efficiency. Transcriptome sequencing can be used to explore specific indicators at a deeper level, such as the content (high and low) and composition of intramuscular fat (IMF) in meat processing, the expression of DEG-related hormones, the regulation of bile acid concentration on fat precipitation, and the regulation of growth and meat quality properties in cattle and sheep. During the lactation stage of ruminants, transcriptome sequencing is used to screen for differentially expressed genes in blood tissue, which can identify candidate functional genes for milk production traits. Transcriptome sequencing can detect genes with low expression levels, identify new gene transcripts and alternative splicing events, detect and analyze the biological regulatory mechanisms of the body, reveal differences in gene expression levels during breeding, and reveal the interaction between hosts and pathogens. This sequencing technology can also help understand the immunobiological situation during infection. This paper reviews the current status of transcriptome sequencing and the application of transcriptome sequencing technology in ruminants, with a view to providing theoretical reference and basis for the better application of transcriptome sequencing technology in ruminant research.

## 1 Introduction

Transcriptomics is an approach to study gene expression and gene regulation by altering mRNA, protein, and metabolite expression, or transcript abundance, and is one of the most widely used techniques in basic research, clinical diagnostics, and drug development (1). Among these, transcriptome sequencing (RNA-seq) can be used to study many aspects of RNA biology, including single-cell gene expression, the translatome, the RNA structural group, and spatial transcriptomics (2). In recent years, with the rapid development of RNA-seq, it has been widely used in animal husbandry. Ruminants are one of the most ecologically important groups of herbivores, exhibiting diverse morphologies (e.g., different body sizes, dentition) and being highly adaptable to a variety of ecological environments (e.g., polar regions, the Tibetan Plateau, desert steppes, and tropical rainforests) (3). Among ruminants, cattle and sheep have high economic and nutritional values. So far, many scholars at home and abroad have used RNA-seq to study ruminants such as cattle and sheep, mainly through this method to reveal potential candidate genes and meat quality, milk quality, skeletal muscle development, fat deposition, reproduction and breeding, and other growth-related functions and disease causative mechanisms. This paper describes the latest progress in the application of transcriptome sequencing technology in ruminants, with a view to providing theoretical references for subsequent studies on ruminant transcriptomics.

## 2 RNA-seq

**Figure 1:**
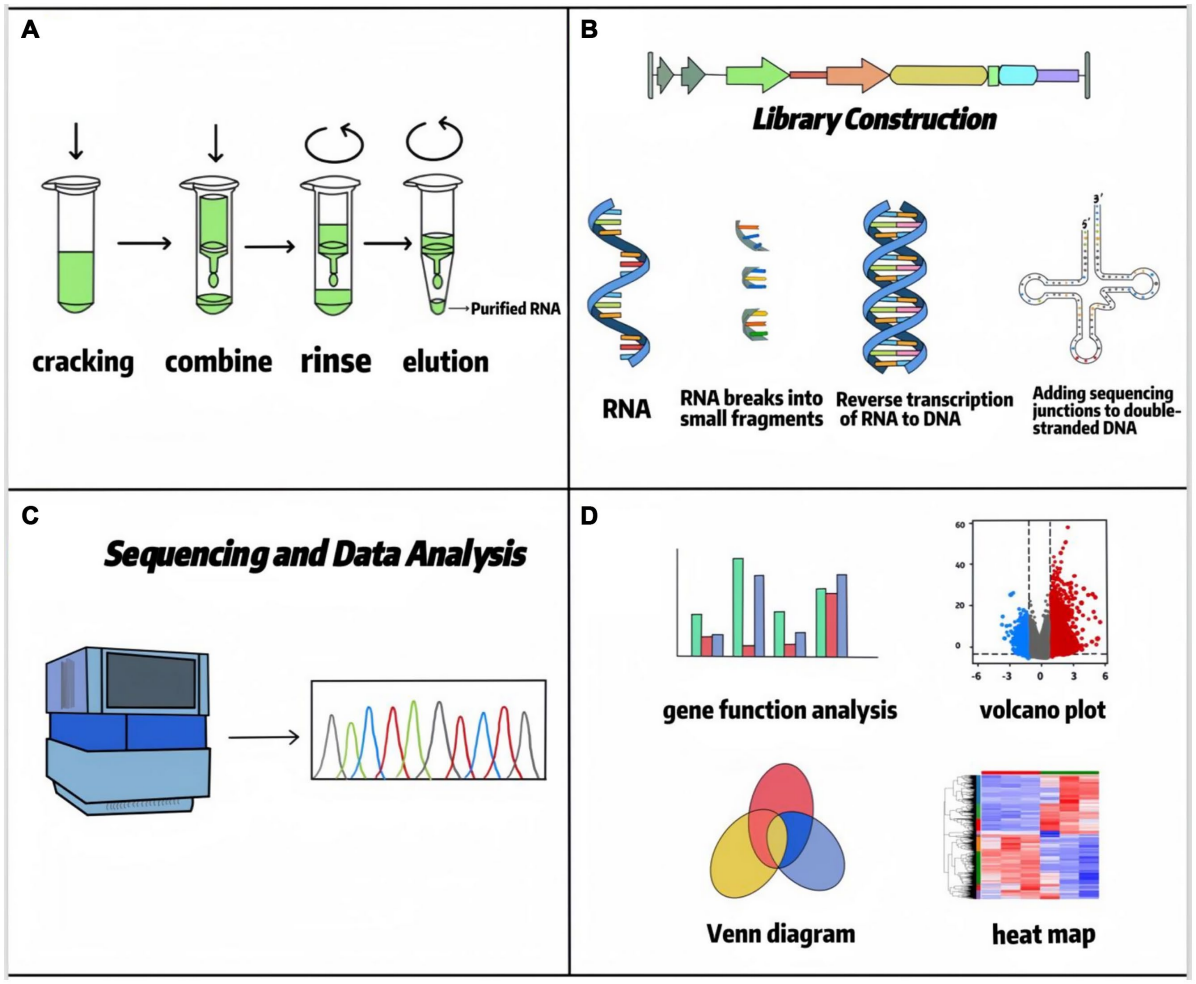
RNA seq flowchart.

In 1970, high-throughput sequencing technology began to be applied in cellular and tissue transcripts, and since then, transcriptome sequencing appeared and has been widely used in various types of production practices (4). Transcriptome sequencing (RNA-seq) represents a comprehensive and efficient approach for capturing the complete set of transcripts within a specific organ or tissue under defined conditions. Subsequent bioinformatic analysis, particularly differential expression analysis (DEA), enables precise interrogation of sequencing data. DEA forms the core of transcriptome research. It employs statistical models, such as the *negative binomial distribution* utilized by tools like *DESeq2*, to compare gene expression levels across distinct experimental conditions (e.g., treatment versus control groups). This process identifies genes exhibiting significant expression changes, commonly defined by thresholds of |log2 (fold change) | > 1 and a *false discovery rate* (FDR) < 0.05. This analysis is crucial for revealing key functional genes, such as identifying candidate genes associated with traits like feed efficiency in ruminant research. RNA-seq serves as a critical link between phenotype and the underlying DNA coding information. The standard RNA-seq data analysis workflow typically includes: raw data quality control and alignment (utilizing tools like *FastQC* for quality assessment and *STAR* or *HISAT2* for reference genome alignment), gene/transcript quantification (generating expression matrices using tools like *HTSeq* or *featureCounts*), differential expression analysis (employing software such as *DESeq2* or *edgeR*), and functional annotation [conducting pathway enrichment analysis using databases such as *Gene Ontology* (GO) and *Kyoto Encyclopedia of Genes and Genomes* (KEGG)] (5, 6). These methodologies allow RNA-seq to characterize average expression profiles within cell populations and, through single-cell RNA sequencing (scRNA-seq), to resolve cellular heterogeneity. Nowadays, RNA-seq is more mature in determining the level of differential gene/isoform expression and has become one of the most commonly used tools in biology, which has changed our view of the complexity of the transcriptome, which provides new information on transcriptional and post-transcriptional gene regulation. The RNA-seq workflow is divided into RNA extraction, library construction, sequencing, and data analysis (7), as shown in Figure 1. Building upon standard RNA-seq, diverse methodological approaches have emerged based on distinct technical principles and application requirements (summarized in Table 1). These platforms are tailored to specific research objectives. For instance, short-read RNA-seq (e.g., *Illumina*) offers high cost-effectiveness, making it suitable for large-scale population differential expression analyses. Conversely, long-read RNA-seq technologies (e.g., *PacBio* or *Oxford Nanopore*) enable the resolution of complex transcriptional isoforms, proving particularly valuable for investigating intricate interaction mechanisms between rumen microorganisms and their host species (8, 9) (see Table 2). In addition, RNA-seq has facilitated the development of RNA biology, a methodology that can accurately characterize the interactions between transcription and molecules controlling RNA function and can also be used to study many different aspects of RNA biology, i.e., single-cell gene expression, the translatome, and the RNA structuralome. Haque A et al. (10) provided guidelines for the use of single-cell transcriptome sequencing (scRNA-seq) for biomedical and clinical purposes, which lists problems that may be encountered when using scRNA-seq, including protocol selection and biological interpretation. Currently, scRNA-seq has been widely used in cancer therapy (11), infectious diseases (12), and regulation of stem cell differentiation (13). The concept of spatial transcriptomics was first introduced by Joakim Lundeberg’s group in 2016, and spatial transcriptomics technology allows gene expression information to be obtained at spatial resolution from intact tissue sections in pristine physiological environments (14). In recent years, spatial transcriptomics has been used to study liver regeneration, organization, and function in hepatocytes and nonparenchymal cells, as well as to understand intercellular variation within and between individual tumors, advancing cancer research and diagnosis (15–17).

**Table 1:** Common applications of RNA-seq technologies.

Category	Technology	Principle	Key features	Typical applications
Sequencing-based	Short-read RNA-seq	Fragmentation of RNA/cDNA into 50–300 bp segments (Illumina platform)	Low cost, high throughput. Requires assembly. Splice variant resolution algorithm-dependent	Gene expression quantification, differential analysis (Bulk/scRNA-seq), simple transcript identification
	Long-read RNA-seq	Direct sequencing of full-length RNA/cDNA (>10 kb; PacBio/ONT platforms)	No assembly required. Accurate isoform/fusion gene detection. Single-cell isoform analysis (e.g., scISO-seq)	Complex transcriptome analysis (splicing, lncRNA), novel transcript discovery
Non-sequencing	Microarray	Hybridization of cDNA to fixed probes with fluorescence quantification	Limited to known genes. Low cost but low sensitivity. Probe-dependent.	Clinical gene profiling (e.g., tumor classification arrays)
	qPCR/ddPCR	Target-specific primer amplification for RNA quantification	Ultra-sensitive (single-molecule). Limited to small gene sets.	RNA-seq validation, low-abundance transcript detection
	Nanopore direct RNA-seq	Native RNA sequencing (no reverse transcription; Oxford Nanopore)	Preserves RNA modifications (e.g., m^6^A). Real-time monitoring. Requires error correction.	RNA modification analysis, transcriptional dynamics
	Spatial transcriptomics	In situ hybridization (ISH) or antibody-based RNA mapping (e.g., MERFISH)	Single-cell spatial resolution. No single-cell isolation needed.	Tumor microenvironment mapping, spatiotemporal gene expression in embryogenesis
Advanced methods	Epitranscriptomics	meRIP-seq (antibody enrichment) or direct modification detection (Nanopore)	Profiles RNA modifications (m^6^A, m^5^C).	Disease mechanisms (e.g., cancer, neurodegeneration)
	Single-cell multi-omics	CITE-seq (transcriptome + surface proteins) SHARE-seq (transcriptome + ATAC)	Multi-modal data integration. Deciphers cellular states.	Tumor immunology, cell fate determination

**Table 2:** Characteristics, applications and costs of short-read vs. long-read RNA-seq in ruminant studies.

Feature	Short-read RNA-seq (Illumina)	Long-read RNA-seq (PacBio/Oxford nanopore)
Read length	50-300 bp (typically 75–150 bp)	1,000-100,000 + bp (full-length transcripts)
Throughput	Extremely high (millions of reads per run)	Moderate (PacBio), High (Nanopore R10.4+)
Accuracy	High base precision (Q30 > 99.9%)	Moderate (PacBio HiFi: >99.9%; Nanopore: ~95–98%)
Key advantages	Low cost for large population studies High sensitivity for gene expression detection Mature bioinformatics tools	Captures full-length isoforms Direct RNA sequencing (Nanopore) Resolves complex gene families/structural variants
Limitations	Requires transcriptome assembly Ambiguity in isoform resolution GC bias	Higher raw error rates Lower throughput (PacBio) High computational requirements
Applications in ruminants	Differential gene expression (e.g., lactation, disease) Large-scale eQTL studies miRNA/small RNA analysis Population transcriptomics	Alternative splicing analysis (e.g., immune response, stress) Fusion gene detection Allele-specific expression Novel isoform discovery
Cost per sample	$20–100 (bulk RNA-seq)	$100–500 + (depending on sequencing depth)
Recommended scenarios	Large population studies Gene expression quantification Well-annotated genomes	Isoform-level biology research Poorly annotated genomic regions Structural variant detection

### 2.1 Application of RNA-seq in the diagnosis of genetic diseases

The widespread adoption of high-throughput sequencing has substantially increased the identification of variants of unknown significance (VUS) in clinical diagnostics. According to established clinical guidelines, such as those from the *American College of Medical Genetics and Genomics* (ACMG), VUS cannot be used directly for clinical decision-making. RNA-seq serves as a critical supplement to genome sequencing, enabling effective analysis of VUS and improving the diagnostic yield for genetic disorders (18). This utility is demonstrated through several key capabilities: Detecting aberrant gene expression levels (e.g., downregulation), indicative of potential loss-of-function mechanisms; Accurately identifying abnormal mRNA splicing events, including exon skipping, intron retention, and the creation of novel splice sites, even capturing non-canonical splicing variations potentially missed by DNA sequencing alone; Revealing allele-specific expression (ASE), providing evidence for the functional impact of regulatory, splicing, or nonsense-mediated decay (NMD) variants; Enabling efficient and sensitive detection of fusion gene transcripts, particularly in scenarios where DNA sequencing may be limited (19–22). Consequently, RNA-seq plays a vital role in genetic disease diagnosis by verifying the pathogenic consequences of DNA variants, uncovering pathogenic variants overlooked by standard DNA sequencing (such as splicing defects and fusion genes), and providing essential evidence for interpreting VUS to achieve definitive diagnoses.

### 2.2 Application of RNA-seq in biologics production efficiency and quality control

RNA-seq enhances production efficiency and quality control during cell line development for biologics manufacturing (23). This technology enables comprehensive characterization of gene expression dynamics across the host cell system, clonal selection, and culture processes. It identifies key genes influencing critical attributes, such as *sialyltransferase* expression impacting cell growth, product titers (e.g., monoclonal antibody yield), and metabolic phenotypes (e.g., glycosylation profiles). These insights guide culture medium optimization and targeted cell engineering strategies (24). Concurrently, RNA-seq provides sensitive quality control by detecting low-frequency variants within the gene of interest (GOI), including Single nucleotide variations (SNVs, down to 0.1% allele frequency); Insertions and deletions (indels); Aberrant splicing events (e.g., *intron retention*, *exon skipping*). Integration with mass spectrometry facilitates resolving sources of product impurities, such as truncated heavy chains, ensuring the accuracy of the therapeutic protein’s primary structure (25). Furthermore, RNA-seq efficiently detects fusion transcripts often missed by DNA sequencing and analyzes regulatory networks involving non-coding RNAs (ncRNAs). For instance: *microRNAs* (miRNAs) can target secretion pathway genes to modulate production; *Long non-coding RNAs* (lncRNAs) exhibit expression patterns correlating with cell proliferation and product stability. Finally, single-cell RNA sequencing (scRNA-seq) resolves transcriptional heterogeneity within clonal populations. This capability identifies sources of production instability, such as transgene expression attenuation, and provides a basis for screening clones with high phenotypic consistency (26).

### 2.3 Application of scRNA-seq in multidimensional integration

As a cornerstone of single-cell multi-omics, single-cell RNA sequencing (scRNA-seq) elucidates intrinsic cellular heterogeneity by resolving gene expression profiles at the single-cell level. The workflow encompasses single-cell suspension preparation, mRNA capture, reverse transcription, and nucleic acid amplification. Subsequent bioinformatic analysis, typically performed using *R*-based tools like *Seurat* or *Python* frameworks such as *Scanpy* (utilizing the *AnnData* structure) (27), includes: Data quality control and filtering; Selection of highly variable genes (HVGs); Dimensionality reduction (employing techniques like *principal component analysis* (PCA), *Uniform Manifold Approximation and Projection* (UMAP), or *t-distributed Stochastic Neighbor Embedding* (t-SNE)); Cell clustering and annotation; Differential expression gene (DEG) analysis; Functional pathway enrichment analysis (utilizing resources like *Gene Ontology* (GO) and *Kyoto Encyclopedia of Genes and Genomes* (KEGG)); Cell–cell interaction inference; Cellular trajectory inference (28). While scRNA-seq enables identification of genes influencing cell growth and product titers for biopharmaceutical process optimization, it faces challenges including high cost and stringent sample viability requirements. Advanced multidimensional integration strategies enhance scRNA-seq capabilities: Temporal Dynamics: Integration with metabolic labels (e.g., 4-thiouridine (4SU), 5-ethynyluridine (EU)) or fluorescent reporters enables tracking transcriptional changes over time; Spatial Context: Spatial transcriptomics technologies overcome the spatial limitation of standard scRNA-seq by mapping gene expression directly within tissue architecture. This is achieved through methods like *in situ hybridization* (ISH), *in situ sequencing* (ISS), or spatial barcoding (29–32); Multi-omics Integration: Coupling scRNA-seq with genomics (e.g., scATAC-seq for chromatin accessibility), epigenomics, and proteomics (e.g., *CITE-seq* for surface protein detection) provides a comprehensive view of cellular state and function. This integrated approach demonstrates significant value in revealing heterogeneity, discovering novel therapeutic targets, and advancing research in tumor immunology, developmental biology, and disease mechanisms, thereby promoting multidimensional biological investigation from gene expression to functional understanding (33).

## 3 Application of RNA-seq in ruminants

### 3.1 Application in meat quality

In the past, meat production has been improved mainly by screening strong individuals in the population for breeding, a method that is inefficient and does not allow for the continued inheritance of good genes after intergenerational or several generations of breeding. RNA-seq can be used to optimize breeds by screening genes and is suitable for the selection of various expressed genes related to meat quality among different species in the livestock industry (34). In recent years, RNA-seq assays have been used in ruminants in applications involving the genetic mechanisms of m6A methylation, muscle quality under specific conditions, and muscle mass under breed self-differentiation conditions. In the molecular mechanisms involved in muscle growth and development, previous experiments have used m6A seq, MeRIP seq, and RNA seq high-throughput sequencing to perform bioinformatics analysis on the longest dorsal muscle of different cattle and sheep (35–37). It mainly contains GO and KEGG pathway enrichment analysis, identification of differentially expressed genes and differentially expressed lncRNAs, as well as detection of the expression levels of muscle-related marker genes and methylation-related enzymes using techniques such as qRT-PCR, Western blot, and LC–MS/MS for auxiliary validation. These assays allow the identification of key genes involved in the development of muscle growth and m6A modifications, such as those involved in biological processes such as skeletal muscle contraction, steroid biosynthesis processes, redox processes, the PPAR pathway, and fatty acid metabolism. Purebred cattle and sheep m6A modifications are mainly enriched in the 3′-UTR region and the CDS region. The 3’ UTR is immediately adjacent to the downstream region of the CDS, which is transcribed but not translated with the gene, and this region is the main mediator of mRNA stability and translation. The hybrid yak (*Bos grunniens*) was mainly enriched in muscle-related pathways (Wnt signaling pathway and MAPK signaling pathway) and plateau-acclimation-related pathways (HIF-1 signaling pathway). The m6A abundance is positively correlated with gene expression levels. There is a gap in the current experiments to assess the long-term effects of m6A modification on muscle growth and development in ruminants, and RNA-seq-based m6A-modified meat improvement methods could be developed in future studies.

Physiological differences in meat tenderness in ruminants are variables in addition to their own genetic regulation. Environmental stresses and high-temperature stress can also lead to endocrine disruption, abnormal nutrient metabolism, and changes in body tissue composition (38–40). RNA-seq enables gene ontology and KEGG pathway enrichment analysis to identify biological processes and signaling pathways associated with physiological responses and meat quality. Stress response in sheep manifests as increased plasma adrenaline concentration, decreased muscle glycogen concentration, elevated meat shear force, and reduced cooking loss. The anti-apoptotic function of *heat shock protein* (HSP) genes (*HSPA12A*, *HSPB8*) and the metabolic regulation mediated by *AMP-activated protein kinase* (AMPK) genes represent core mechanisms underlying this stress response. These genes serve as molecular markers for meat stress injury, providing a theoretical foundation for mitigation strategies through genetic breeding or nutritional interventions (e.g., AMPK activators) (41). HSP genes (e.g., *HSPA12A*, *HSPB8*, *HSP70*, and *HSP90*) exhibit significant upregulation under stress and maintain cellular homeostasis via molecular chaperone activity, though their regulatory mechanisms differ across species (direct transcriptional upregulation in goats versus negative regulation by miRNAs, such as miR-2450, in cattle). Transcriptomic changes involve pathways related to stress response, apoptosis, protein hydrolysis, and energy metabolism (e.g., AMPK, *JAK–STAT*, and PI3K-Akt), demonstrating how stress impacts animal physiology and meat quality through multi-gene networks (42). The related SLC2A4 gene serves as a key metabolic regulator of granulosa cells in response to heat stress by optimizing sugar metabolism and energy supply. During heat stress, granulosa cells significantly upregulate *SLC2A4* expression to optimize glucose metabolism and energy supply. This enhanced glucose transmembrane transport provides critical substrates for antioxidant defense, cellular repair, and proliferation recovery (43). RNA-seq can detect biological processes involved in stress, such as energy metabolism, apoptosis, and stress response. The important intracellular energy-regulated signaling pathways AMPK and autophagy pathways are enriched in stress treatments, and physiological stress responses can negatively affect goat meat quality. Clinically, there are herbal extracts that can alleviate stress. Chen H et al. used RNA-seq assays and found that the addition of geraniol to concentrates activated the PPARγ signaling pathway to regulate lipid metabolism and improve the flavor of beef (44). Growth, development, and meat quality of ruminants are not only influenced by external factors (e.g., genetics, environment, feeding management, etc.) but also regulated by several genes. RNA-seq technology in the study of meat quality can comprehensively and high-throughputly detect differences in gene expression levels, revealing the molecular mechanisms of meat quality regulation, and at the same time, it can accurately and reliably screen out functional genes related to meat quality, providing a theoretical basis for meat quality improvement. It is expected to provide biomarker identification for animal selection and improvement of cattle and sheep production.

### 3.2 Application in skeletal muscle

Skeletal muscle is one of the main sites of metabolism, and factors such as survival environment and different growth intervals in ruminants produce different feedbacks on the metabolic cycle during growth and development (45, 46). In the development of the yak industry in the highland region, the growth rate and meat production have been maintained at a low level due to environmental problems. There were significant differences between oxidized (biceps femoris, BF) and glycolyzed (external abdominal oblique, EOA) muscles in different yaks at the transcriptome level, and between chromatin openness to regulate muscle growth, and between the effects of different feeding practices on growth performance, meat quality, and liver function in yaks (47–49). There are also effective studies from the perspective of chromatin accessibility, such as Li J et al. (50), who used ATAC-seq and RNA-seq techniques to analyze and look for differences in chromatin accessibility and gene expression levels during yak skeletal muscle development. These differences are mainly enriched in functions and pathways such as cell proliferation, regulation of biological processes, and transcriptional regulatory activity. The more important cells in skeletal muscle growth and regeneration are skeletal muscle satellite cells (SMSCs), and changes in chromatin accessibility play a significant role in the myoblast differentiation of SMSCs. Myoblast differentiation is a complex process involving the synergistic action of multiple genes and signaling pathways. Some key pathways associated with the proliferation and differentiation of sheep SMSCs, such as the PI3K-Akt signaling pathway, the p53 signaling pathway, the Hippo signaling pathway, myocyte cytoskeleton regulation, and the calcium signaling pathway, were detected by ATAC-seq and RNA-seq (51). Beef quality traits such as tenderness, juiciness, and marbling are usually characterized by the inheritance of quantitative traits, and the degree of genetic control is usually low. The study of cytoskeletal structure in the study of genetic mechanisms of beef quality traits can be positioned in the field of genetics and molecular biology of meat quality traits, which is an important direction in the study of meat quality traits. Cytoskeletal and transmembrane-anchored genes and pathways can be identified in expression association, DE, and gene enrichment analyses, and cytoskeletal proteins and transmembrane-anchored molecules can influence sarcomeres by allowing cytoskeletal interactions with myocyte and organelle membranes, contributing to the maintenance of cytoskeletal structure and architecture after death (52). Differentially expressed genes and miRNAs are also present in the muscle tissues of goat fetuses at different developmental intervals, and in general, the expression levels of muscle differentiation marker genes correlate with the degree of muscle differentiation. The expression profile analysis of miRNA and mRNA can identify certain miRNAs that co-regulate muscle differentiation with target genes, such as chi-miR-129-5p, chi-miR-433, and chi-miR-24-3p, which co-regulate differentiation with genes such as “calcium binding” and “cell adhesion” (53). The miRNA and mRNA expression characteristics and their interactions during skeletal muscle differentiation can be revealed by RNA-Seq technology, providing a new molecular perspective for understanding the mechanism of skeletal muscle differentiation.

### 3.3 Application in fat deposition

Intramuscular fat (IMF) is the fact that accumulates between muscle bundles or within muscle cells, and its content significantly affects the taste, tenderness, and flavor of meat products, which is a crucial economic characteristic in meat production and processing (54, 55). In sheep, fat is mainly deposited in specific areas such as the tail or rump. Bakhtiarizadeh MR et al. (56) used RNA-seq to identify single nucleotide polymorphisms (SNPs) associated with 18 and 52 genes involved in the shape of the fat tail in two Iranian sheep breeds (Lori-Bakhtiari and Zel). These SNPs are located in regions of genes that are fat-tailed or fat-metabolizing functions, and the SNPs are also localized within QTL regions associated with adiposity. The CDS2 gene in Lori-Bakhtiari and PCDH9 gene in Zel were more affected, where some genes related to fatty acid oxidation, such as the PPAR signaling pathway and fatty acid oxidation-related genes, were present in the Zel variety. Intramuscular fat (IMF) deposition is one of the most important factors affecting meat quality and is closely related to the expression of carnitine palmitoyltransferase 1A (CPT1A), which promotes the transfer of long-chain fatty acids (LCFA) to mitochondria (57). Some goat intramuscular precursor adipocyte differentiation can also be identified using RNA-seq to characterize temporal expression profiles during the process, and RNA-seq identified changes in CPT1A expression in goat intramuscular precursor adipocytes that may reconfigure the lipid distribution between intracellular triglyceride deposition and cell proliferation. CPT1A promotes the proliferation of goat adipocytes through the MAPK signaling pathway. After CPT1A knockdown, DEGs were mainly enriched in the MAPK signaling pathway. The MAPK signaling pathway inhibitor PD169316 inhibited cell proliferation in CPT1A-overexpressing adipocytes (58). RNA-seq has screened for miR-433, which is associated with thermogenesis in brown adipose tissue, in the analysis of miRNA expression differences between brown adipose tissue and white adipose tissue (WAT) in goats. MiR-433 mainly inhibits lipid droplet accumulation and thermogenesis in brown adipocytes and suppresses their differentiation by targeting the MAPK8 gene (59). The regulation of adipogenesis can be systematically investigated through overexpression cell models combined with RNA sequencing. *Peroxisome proliferator-activated receptor gamma* (PPARG) serves as a central hub in lipid metabolism. Elucidating its mechanisms advances our understanding of obesity and metabolic disorders while providing critical targets for enhancing meat quality in livestock production. As a master transcriptional regulator, PPARG binds promoter regions of target genes (e.g., stearoyl-CoA desaturase-1 [*SCD1*]), orchestrating fatty acid desaturation and *triglyceride* (TG) synthesis to modulate fat deposition. This pathway is subject to multi-level regulation: Direct transcriptional control by coding genes (e.g.*, TUSC5*), indirect modulation via non-coding RNAs (e.g.*, lncBNIP3*-mediated signaling pathway activation). Targeted manipulation of the PPARG/*SCD1* axis offers promising strategies for meat quality improvement: Genetic approaches: *TUSC5* isoform screening via CRISPR-based editing; Epigenetic regulation: *lncBNIP3* activation to fine-tune expression; Nutritional management: Optimized finishing diets to promote intramuscular fat deposition. These interventions demonstrate significant potential for enhancing beef marbling and sensory attributes (60–62). MiRNA-combined metabolomics also allows for the detection of different lipid-forming conditions, which are enriched between perirenal adipocytes (PRA) and intramuscular adipocytes (IMA) in Qinchuan cattle (63). IMA contained more unsaturated fatty acids than PRA, with IMA producing more in the early stages of adipogenesis and PRA having greater adipogenic capacity in terminal differentiation. The two differentially expressed genes were positively correlated with arachidonic, phosphatidylcholine, phosphatidylethanolamine, and sphingomyelin content. Huang J et al. (64) analyzed potential genes characterizing IMF deposition in buffaloes by transcriptome sequencing. 1,566 mRNAs were expressed in adipose tissues in the mRNA expression pattern, and the results of RT-qPCR analysis and gain-of-function experiments confirmed that phosphoenolpyruvate carboxykinase 1 (PCK1) expression was positively correlated with IMF in buffaloes. The activity of the buffalo promoter PCK1 was confirmed to be higher than that of bovine adipocytes. Investigation of key candidate genes and metabolic pathways regulating adipogenesis reveals that both *leptin* (*LEP*) and fatty acid-binding protein 4 (*FABP4*) significantly influence lipid deposition. *LEP* expression exhibits a positive correlation with adiposity, while *FABP4*—a central mediator of fatty acid transport—modulates intramuscular fat content and unsaturated fatty acid composition. Both genes function as downstream effectors in the *peroxisome proliferator-activated receptor gamma* (PPARγ) signaling pathway, which integrates lipid metabolism, energy homeostasis, and inflammatory responses during adipocyte differentiation. Through transcriptional regulators (*PPARGC1A*, *RXRA*), *LEP* and *FABP4* coordinate: Fatty acid biosynthesis; Lipid droplet homeostasis; while exhibiting negative correlations with lipolytic genes (e.g., *lipase E* [*LIPE*], *perilipin 1* [*PLIN1*]). Tissue-specific expression profiling demonstrates significantly higher transcript levels of both genes in subcutaneous fat and mammary fat pad (MFP) compared to visceral fat or mammary gland parenchyma (PAR). Elevated *FABP4* expression in BF underscores its pivotal role in subcutaneous adipogenesis. Interbreed analyses reveal: Differential *FABP4* expression between Shandong black and Luxi cattle correlates with meat fatty acid profiles, DEG studies in Angus × Simmental crosses indicate breeding value for marbling traits Integrated validation through RNA-seq, qPCR, and western blotting confirms conserved mechanisms of *LEP* and *FABP4* in bovine lipid metabolism, providing molecular targets for beef quality enhancement (65–68). Ruminants with a greater proportion of muscle growth than fat deposition can provide consumers with a higher quality diet. Bile acids regulate the integrated mechanisms of fat distribution in ruminants, increase lamb meat production, and reduce subcutaneous and tail fat deposition (69). It has been shown that adipocyte differentiation and adipose metabolism are tightly regulated by a variety of transcription factors, including sterol regulatory element-binding proteins, CCAAT/enhancer-binding proteins, and peroxisome proliferator-activated receptor gamma. miRNAs can play a key role in adipocyte differentiation and fat metabolism. Comparative analysis of gene expression profiles between different samples by RNA-seq allowed identification of key genes and regulatory networks associated with fat deposition. This will allow a deeper understanding of the genetic mechanisms involved in fat deposition in ruminants, as well as a comparative analysis of genetic differences between breeds to find single nucleotide polymorphisms (SNPs) associated with differences in fat.

### 3.4 Application in lactation

Milk fat content, as an important indicator for evaluating milk quality, directly determines the nutrition and flavor of milk. Milk production in mammalian mammary glands is influenced by various functional genes. Therefore, analyzing the molecular mechanisms of mammary gland development and lactation is essential for improving milk production and quality in dairy cows. Transcriptomic studies of caprine mammary tissue reveal that diacylglycerol O-acyltransferase 1 (*DGAT1*) modulates lipid metabolism through dual mechanisms: inhibiting triglyceride synthesis/fatty acid esterification while upregulating lipolysis genes. This coordinated regulation governs lipid accumulation and monounsaturated fatty acid production. In bovines, *DGAT1* expression correlates significantly with milk yield. As the rate-limiting enzyme in triglyceride synthesis, it directly influences milk fat composition. Its central position in lipid metabolism further suggests indirect modulation of the mammary immune microenvironment, providing multidimensional insights into milk fat synthesis genetics and dairy cow health (70, 71). The *growth hormone receptor* (*GHR*) gene is equally pivotal in ruminant development and lactation. In Chios dairy sheep, *GHR* resides within a milk production quantitative trait locus (QTL), regulating lactation through growth hormone signaling. Its hepatic expression pattern influences milk composition via systemic metabolism. Beef cattle studies demonstrate coordinated reduction of *GHR* and insulin-like growth factor 1 (*IGF1*) mRNA during winter gestation in high-forage regimens, corresponding with serum IGF-I fluctuations. Crossbred cattle exhibit enhanced metabolic flexibility during energy transitions (72, 73). *DGAT1* and *GHR* represent complementary regulatory axes, *DGAT1* operates through lipid metabolic machinery (milk fat synthesis), *GHR* functions via endocrine signaling (growth/lactation performance). These genes serve as molecular markers for: Milk quality improvement (fat content/profile); Health-production synergy optimization; Hybrid vigor exploitation in breeding programs. Long-stranded noncoding RNAs (lncRNAs) also play a key role in mammary gland development and breast cancer biology (74). Chen Yidan (75) used second-generation transcriptome sequencing to detect differentially expressed genes in the blood tissues of high- and low-producing dairy cows and screened a total of 12 candidate functional genes affecting milk-producing traits, of which nine contained mutant alleles (ASS1, DEFB4A, UPP1, GGT1, HP, MGAM, LTF, MMP9, and PGLYRP1), which were found in a higher mutation frequency in the high-yielding group and a lower frequency of genetically variable alleles in the low-yielding group. Little is known about the role of lncRNAs in bovine lactation in current studies. In order to characterize the role of lncRNAs in bovine lactation, Wang Y (76) detected lncRNAs in mammary tissues of dairy cows at 7 d pre-partum and 30 d post-partum by using RNA-Seq technology. Ninety-six lncRNAs were significantly differentially expressed between the two stages, and the target genes were mainly focused on ECM-receptor interactions, the Jak–STAT signaling pathway, the PI3K-Akt signaling pathway, and the TGF-beta signaling pathway, which may inform the expression profiles and characterization of the pathways in both non-lactating and early lactation breast tissues. In lactation studies on breed-specific goats, suppressor of cytokine signaling 3 (SOCS3) is closely related to lipid metabolism in dairy goats and is a key signaling molecule that regulates milk synthesis in domestic animals. Song N (77) screened key downstream genes associated with SOCS3-regulated lipid synthesis in goat mammary epithelial cells (GMEC) by RNA-seq and identified a total of 430 differentially expressed genes, including 226 down-regulated and 204 up-regulated genes. Among them, STAT2, FOXO6, BCL2, MMP11, MMP13, and CD40 are key regulatory genes involved in lipid metabolism. Farhadian M (78) In order to continue to investigate the underlying molecular mechanisms of the mammary lactation process, Ghezel sheep were analyzed by transcriptome sequencing technology in two stages of lactation, pre- and post-peak lactation. Seventy-five differentially expressed genes were screened, mainly enriched in pathways such as metabolic processes and oxidative phosphorylation. Gene network analysis showed that the peroxisome proliferator-activated receptor (PPAR) signaling pathway, oxidative phosphorylation, and metabolic pathways play important roles in milk production. Among them, genes related to fat metabolism were significantly downregulated during the post-peak milk production phase. In summary, these findings offer novel insights into the regulatory architecture of milk fat metabolism in ruminants, unveiling molecular targets with significant potential for enhancing dairy product quality—including strategic development of reduced-fat dairy lines—through precision modulation of lipid biosynthesis pathways. RNA-seq technology for mining functional genes and genetic variants for milk production traits in cows and goats can identify candidate genes for the prevention of inflammation and disease in mammary tissues, and at the same time, by comparing with the QTL databases for milk production traits, it can be further mined for functional genes related to lactation traits, which can help to discover the key regulatory factors, but it needs to be investigated in more depth.

### 3.5 Application in breeding and reproduction

In reproductive breeding, RNA-seq can detect and analyze the biological regulatory mechanisms of ruminants, which can provide scientific basis and technical support for improving the breeding and reproduction efficiency of cattle and sheep. The pituitary gland can directly regulate reproduction in livestock, and Wan Z et al. (79) performed RNA-Seq in order to characterize transcriptomic differences in the pituitary gland of sheep during the estrous cycle. A total of 3,529 lncRNAs and 16,651 mRNAs were identified in the pituitary gland, of which 144 differentially expressed (DE) lncRNA transcripts were screened in the follicular and luteal phases and 557 DE mRNA transcripts. In addition, GO and KEGG analyses indicated that 39 down-regulated and 22 up-regulated genes interacted with pituitary function and reproduction. These findings provide a genome-wide lncRNA and mRNA expression profile of the sheep pituitary between the follicular and luteal phases, which will help to further investigate the molecular mechanisms of pituitary function. Lu X et al. (80), in order to characterize whether the pituitary gland influences bovine growth by regulating hormone secretion, assayed the levels of six growth-related HPT hormones in the plasma of antelope and Reychon cattle and compared the transcriptomic data of their pituitary glands. This resulted in significant differences in growth hormone, IGF, TSH, thyroxine, triiodothyronine, and insulin content between the 2 varieties, with a total of 175 genes identified as differentially expressed genes (DEGs). Functional association analysis showed that DEGs were mainly involved in transcription and signal transduction processes. Combined with enrichment and protein interaction analyses, eight DEGs (SLC38A1, SLC38A3, DGKH, GNB4, GNAQ, ESR1, NPY, and GAL) were predicted to control the growth of antelope and Reykjavik cattle by affecting the expression of growth-related hormones in the pituitary gland. Huang Y et al. (81) compiled mRNA expression profiles of the pituitary and hypothalamus in Angus cattle at different growth and developmental stages, providing transcriptomic data for the study of the hypothalamic pituitary in Angus cattle. Analyzing 6-, 18-, and 30-month-old Angus cows separately, genes differentially expressed between 18 and 6 months were enriched in the hypothalamus and pituitary for growth, development, and sexual maturation, whereas genes differentially repressed between 30 and 18 months were enriched in lactation promotion. The role of RNA-seq in the pituitary gland is to reveal differences in different genes in the follicular and luteal phases by measuring gene expression levels. Through RNA sequencing analysis, the expression of long-stranded non-coding RNAs and messenger RNAs can be discovered, further helping to reveal the regulatory mechanisms and functions of genes. In actual production, neurons in the brain that control metabolism are connected to reproductive neurons, and factors such as photoperiod, dietary structure, and living environment affect carcass metabolism. Metabolic status influences reproductive status, and differentially expressed genes are activated in multiple pathways, including metabolism, feeding, neurobehavioral, and gustatory sensation. Further functional validation using RNA-Seq can provide insight into the mechanisms by which the hypothalamic–pituitary-gonadal axis regulates reproduction in ruminants such as cattle and sheep. The ability of RNA-seq to detect genes expressed at low levels, identify novel gene transcripts, and variable splicing events can effectively reveal differences in gene expression levels in reproductive breeding and provide useful insights into the genetic background and growth regulation of ruminants.

### 3.6 Application in diseases

Small ruminant lentiviruses (SRLVs) are important pathogens infecting sheep and goats. SRLV infection interferes with innate and acquired host immunity, and the genes associated with resistance and susceptibility to SRLV infection are not fully understood. Olech M et al. (82) used RNA-seq to compare transcriptome changes in SRLV-infected goats and found that a total of 1,434 differentially expressed genes were shown to be involved in immune response, cell cycle regulation, cellular metabolism, and cellular defense mechanisms following SRLV infection. Due to the increasing level of antibiotic resistance in Mycoplasma isolates in recent years and the lack of cell walls in Mycoplasma, it has caused great difficulties in cultivation. Shi et al. (83) used *Mycoplasma pneumoniae* strain N M-151 as a test strain and collected samples from three growth periods of the strain (logarithmic phase, stationary phase, and decline phase) for RNA-seq analysis, thus solving the problem of the difficult culture of *Mycoplasma pneumoniae* in sheep. The optimization of the culture medium was proposed by gene expression level and KEGG enrichment analysis (the ratios of the modified medium: PPLO broth powder 21 g/L, 250 mL/L fermentation bean extract 10 mL/L, glucose 1 g/L, HEPES 5.76 g/L, 4 g/L phenol red 2.5 mL/L, sodium pyruvate at a final concentration of 1 g/L, 30 mg/L serine, 100 U/mL penicillin, erculosis is an important pathogen that infects cattle by aerosol transmission and can form foci characterized by tuberculous granulomas of tissues and organs with caseous, calcific necrosis. and inactivated horse serum 200 mL/L), and culture tests were performed to verify the validity and practicability of RNA-seq. *Bovine tuberculosis*, caused by *Mycobacterium bovis*, is a significant zoonotic pathogen transmitted via aerosols in cattle. Infection manifests pathologically through the formation of tuberculous granulomas featuring caseous necrosis and tissue calcification. During host response stratification, the chemokine gene *CXCL8* emerges as a central mediator of immune reactions to mycobacterial infection. Transcriptomic profiling using the *edgeR* statistical model identified *CXCL8* among significantly upregulated genes. Key findings demonstrate: In 3D bovine lung models, *CXCL8* transcription increased >600-fold (*p* < 0.001) following high-virulence *M. bovis* challenge. This chemokine drives neutrophil chemotaxis and activates type I interferon signaling. *CXCL8* integrates into early inflammatory networks during infection establishment. Notably, persistent *CXCL8* overexpression in peripheral blood of infected cattle forms a stable diagnostic signature, validated as a biomarker suite for tuberculosis surveillance (84, 85). Fang Lichun (86) also used RNA Seq technology to reveal the diversity of physiological status of bovine tuberculosis under different infection conditions, and screened and verified that IL-8, LTA, CRP, BCL2 and CHI3L1 are molecular markers of bovine tuberculosis with diagnostic value, providing a new target for early diagnosis, disease monitoring and treatment of tuberculosis. Itchy is a neurodegenerative disease that belongs to the category of transmissible spongiform encephalopathy (TSE). In the study of infectious itch disease, the function of PRNP gene is closely related to signal pathways such as calcium signaling and cAMP signaling, as well as methylation abnormalities of cell type specific genes, suggesting that it may affect pathological processes through epigenetic regulation of neuronal function and metabolic pathways. Meanwhile, the PRNP gene can regulate the immune response and cell communication related gene expression of peripheral immune tissues (such as retropharyngeal lymph nodes), affecting the susceptibility of sheep to itch disease. Among them, the resistance genotype (R1) may form an early defense barrier by enhancing local immune function (87, 88). In disease applications, RNA-seq technology provides an important tool for studying the mechanisms of ruminant pathogenesis and the plasticity of differential levels of gene expression. Transcriptomic data have an irreplaceable role in targeting the function of ruminant cellular effector molecules and their specificity during addition and inflammatory responses to disease. Analysis of the host transcriptome allows identification of genes associated with ruminant infections and development of potential biomarkers. In addition, RNA-seq helps to resolve immunobiological pathways of infection in cattle and sheep herds, identifying differentially expressed genes and discovering key processes and signaling pathways associated with disease infection. In addition, RNA-seq can reveal host-pathogen interactions and help to understand the immunobiology of the infection process. In the future, with advances in technology and improved methods of analyzing data, RNA-seq could help to identify cattle and sheep with lower viral loads in order to reduce viral transmission and, ultimately, improve the health of animals in affected populations.

## 4 Conclusion and Prospect

Transcriptome sequencing technology is a standard method for selective splicing analysis and provides an effective means to study the relationship between transcriptional regulatory networks and traits. Currently, RNA sequencing (RNA-seq) technology is extensively applied across diverse areas of ruminant research, including meat quality improvement, genetic breeding, and disease prevention and control. By analyzing the expression patterns of key genes (Table 3), we can achieve a more comprehensive understanding of the molecular regulatory mechanisms governing economically important traits in ruminants. RNA-seq is more versatile, focusing on the analysis of functional gene expression and the ability to model changes in disease-inducing variants, and is the link between genomic and proteomic research, with the amount of information generated by the technology allowing for a closer association of genes and traits. Although RNA-seq can unravel fundamental questions such as potential candidate motifs for many ruminants, it is still in the developmental stage and has many challenges to face. Issues such as higher costs, complex data processing, and possible biological bias. Research in the face of RNA-seq requires comprehensive consideration of a variety of factors, including experimental design, sequencing depth, and data analysis methods. In addition, RNA-seq technology itself has certain limitations and errors, such as the relatively complex process of analyzing data extracted from meat, which requires a high level of technical support in bioinformatics, as well as the presence of data errors and noise, and requires more time and resource investment for the processing and interpretation of large-scale data. Therefore, the results need to be treated with caution and validated with other experimental evidence when conducting relevant studies. In recent years, the emerging integration of single-cell and spatial transcriptomics has great potential for clinical applications, study of disease mechanisms, and development of precision therapies. RNA-Seq technology can identify new therapeutic targets in diseases and can determine the spatial localization of rare cell types and cell subpopulations that emerge during diseases. The large amount of data and bioinformatics analysis can be used to mine more potential targets, better characterize the transcription of different cell types in biological tissues, and reveal the heterogeneity of gene expression among cells. The more mature development of RNA-seq in ruminants has not only deepened our understanding of the regulation of gene expression but also provided new perspectives and methods for disease diagnosis and treatment.

**Table 3:** Key genes in ruminant transcriptomics studies.

Gene symbol	Species	Main trait associations	Key transcriptomic findings	Common methods	References
HSP	Cattle, Sheep, Goat	Thermotolerance, Stress Response	Upregulated under heat stress	Short-read (RNA-seq)	(41, 42)
SLC2A4	Cattle, Sheep	Glucose uptake (Muscle/Mammary)	Insulin-responsive glucose transporter, upregulated during acute heat stress	Short-read (RNA-seq)	(43)
PPARG	Cattle, Sheep	Adipogenesis, Marbling	Master regulator of adipocyte differentiation; upregulated in marbled muscle	Short-read (RNA-seq)	(60, 61)
SCD1	Cattle, Goat, Sheep	Milk FA desaturation	Converts SFA to UFA; highly expressed in high-oleic milk	Short-read (RNA-seq)	(60, 62)
FABP4	Cattle, Sheep	Intramuscular fat (IMF)	Fatty acid transporter; biomarker for IMF deposition	Short-read (RNA-seq)	(65, 66)
LEP	Cattle, Sheep	Feed efficiency, Energy balance	Correlates with metabolic status; lower expression in high-efficiency animals	Short-read (RNA-seq)	(65, 67, 68)
DGAT1	Cattle, Goat	Milk fat content, Yield	Rate-limiting enzyme for triglyceride synthesis; positively correlates with milk fat	Short-read (RNA-seq)	(70, 71)
GHR	Cattle	Milk yield, Growth	Mediates growth hormone effects; expression linked to lactation efficiency	Short-read (RNA-seq)	(72, 73)
CXCL8	Cattle, Sheep	Tuberculosis, Immune response	Pro-inflammatory chemokine; activates neutrophil/macrophage bactericidal activity	Short/Long-read	(84, 85)
PRNP	Sheep, Goat	Scrapie resistance/susceptibility	Differential expression between resistant/susceptible genotypes; long-read resolves isoforms	Long-read (Isoform analysis)	(87, 88)

## Glossary

ASE: allele-specific expression
ACMG: American College of Medical Genetics and Genomics
AMPK: AMP-activated protein kinase
BF: biceps femoris
CPT1A: carnitine palmitoyltransferase 1A
DGAT1: diacylglycerol O-acyltransferase 1
DEA: differential expression analysis
DEGs: differentially expressed genes
DE: differentially expressed
EU: ethynyluridine
EOA: external abdominal oblique
FDR: false discovery rate
FABP4: fatty acid-binding protein 4
GOI: gene of interest
GO: Gene Ontology
GLUT4: glucosetransporter4
GMEC: goat mammary epithelial cells
GHR: growth hormone receptor
HSP: heat shock protein
HVGs: highly variable genes
ISH: *In situ* hybridization
ISS: In situ sequencing
InDel: Insertion–Deletion
IGF1: insulin-like growth factor 1
IMA: intramuscular adipocytes
IMF: intramuscular fat
KEGG: Kyoto Encyclopedia of Genes and Genomes
LEP: leptin
LIPE: lipase E
LC–MS/MS: Liquid chromatography tandem mass spectrometry
LCFA: long chain fatty acids
lncRNA: Long stranded noncoding RNAs
LCFA: long-chain fatty acids
MFP: mammary fat pad
miRNA: microRNA
NMD: nonsense-mediated decay
PAR: parenchyma
PLIN1: perilipin 1
PRA: perirenal adipocytes
PPARG: Peroxisome Proliferator-Activated Receptor Gamma
PPAR*γ*: Peroxisome Proliferator-Activated Receptor γ
PPAR: peroxisome proliferator-activated receptor
PCK1: phosphoenolpyruvate carboxykinase 1
PCA: principal component analysis
qRT-PCR: Quantitative Real-time polymerase chain reaction
QTL: quantitative trait locus
SNPs: single nucleotide polymorphisms
SNV: Single Nucleotide Variation
scRNA-seq: single-cell transcriptome sequencing
SMSCs: skeletal muscle satellite cells
SRLV: Small ruminant lentiviruses
SOCS3: suppressor of cytokine signaling 3
t-SNE: t-distributed Stochastic Neighbor Embedding
RNA-seq: transcriptome sequencing
TSE: transmissible spongiform encephalopathy
UMAP: Uniform Manifold Approximation and Projection
VUS: Variants of Unknown Significance
WB: Western blot
WAT: white adipose tissue
